# Estimation of Genomic Breeding Values for Economic Traits in Alxa-Type Cashmere Goats Using Bayesian Methods

**DOI:** 10.3390/ani16203308

**Published:** 2026-10-21

**Authors:** Jie Quan, Tiecheng Wu, Tao Wang, Junyang Liu, Mengyu Guo, Leqi Yi, Bin Liu

**Affiliations:** 1 Inner Mongolia Academy of Agricultural and Animal Husbandry Sciences, Hohhot 010031, China; quanjie_1220@163.com (J.Q.);; 2 National and Local Joint Engineering Research Center for Genetic Resources Evaluation and Breeding Technology of Meat Sheep (Inner Mongolia), Hohhot 010031, China

**Keywords:** cashmere goat, genomic selection, economic traits, Bayesian methods

## Abstract

Conventional breeding has a long generation interval; genomic selection can enable early selection and shorten the breeding process in cashmere goats. This study aimed to estimate genomic breeding values for economic traits in Inner Mongolia (Alxa-type) cashmere goats using Bayesian methods. Whole-genome resequencing at 1× depth was performed on 491 Alxa-type cashmere goats (40 males and 451 females), and genotypes were imputed to 10× using a reference panel with an average imputation accuracy of 97.90%. Fixed effects were determined by analysis of variance, and breeding values for body weight, cashmere fiber diameter, and cashmere yield were estimated using Bayes A, Bayes B, and Bayes C; prediction accuracy and unbiasedness were evaluated by five-fold cross-validation. Measurement year and group affected all three traits highly significantly (*p* < 0.01); sex affected body weight and cashmere yield highly significantly (*p* < 0.01) but no significant effect on cashmere fiber diameter (*p* > 0.05); and maternal age had a significant effect on body weight (*p* < 0.05), but no significant effect on cashmere fiber diameter or cashmere yield (*p* > 0.05); group had a highly significant effect on all three traits (*p* < 0.01). Breeding values were estimated using Bayes A, Bayes B, and Bayes C, with five-fold cross-validation, and the three methods performed comparably overall in this population.

## Simple Summary

Cashmere goats are economically important in Inner Mongolia for both meat and cashmere production. Traditional phenotype-based breeding is slow and imprecise, whereas genomic selection uses genome-wide markers to predict breeding values early in life, thereby accelerating genetic improvement. In this study, we sequenced 491 Alxa-type cashmere goats at low depth (1×) and imputed their genotypes to 10× coverage using a high-quality reference panel (average imputation accuracy of 97.90%). We then compared three Bayesian methods—Bayes A, Bayes B, and Bayes C—for predicting breeding values of body weight, cashmere fiber diameter, and cashmere yield. All three methods yielded comparable prediction accuracies. These findings provide practical guidance for implementing genomic selection in Alxa cashmere goat breeding programs.

## 1. Introduction

The Inner Mongolia (Alxa-type) cashmere goat is an important dual-purpose breed in Inner Mongolia, valued mainly for its high cashmere yield, fine cashmere fiber diameter, and good meat performance. With the expansion of intensive production and growing market demand, body weight, cashmere fiber diameter, and cashmere yield have become increasingly important traits for evaluating meat performance and economic value. Body weight directly affects meat output, cashmere yield directly determines economic returns, and cashmere fiber diameter governs the quality grade and market value of the fiber. Studies have shown complex interrelationships among these traits: body weight is positively correlated with cashmere yield and fiber diameter, and cashmere yield is also positively correlated with fiber diameter [1,2,3]. Therefore, one of the core challenges in cashmere goat breeding is to increase cashmere yield and body weight while maintaining fiber fineness.

Traditional selection breeding relies mainly on phenotypic information and pedigree relationships. Although it has improved breeding efficiency to some extent, its long generation interval, limited prediction accuracy, and high requirements for phenotypic recording make it difficult to meet the need for rapid genetic improvement. Genomic selection, proposed by Meuwissen et al., is a genetic evaluation approach that estimates individual genomic breeding values from genome-wide marker information and has become one of the core technologies for genetic improvement in livestock [4,5,6]. Among these methods, Bayesian models are a class of probabilistic models built on Bayesian statistical theory. Rooted in Bayes’ theorem, they integrate prior distributions of parameters with the likelihood of observed data to infer the posterior distributions of unknown parameters, thereby better capturing the genetic effects of quantitative trait loci [7]. Bayes A assumes that each SNP has a distinct effect variance drawn from an inverse chi-square distribution. Bayes B builds on Bayes A by constructing a sparse model in which only a proportion (1 − π) of SNPs have non-zero effects, with the non-zero effect variances following an inverse chi-square distribution and the remaining proportion π having zero effects. Bayes C, proposed by Habier et al. in 2011, also uses a mixture distribution but assumes equal variance across the non-zero effects, otherwise being the same as Bayes B [8]. These methods usually show greater flexibility and higher prediction accuracy when handling traits influenced by a few major genes together with many small-effect polygenes [4,7,9,10]. These advantages have been corroborated in genomic evaluation practices for livestock and poultry. In laying hens, Wolc et al. (2016) demonstrated that Bayes B had greater power to detect large-effect QTLs for egg weight, yielding genomic predictions that were both more accurate and more persistent than those of GBLUP [11]; In Inner Mongolian cashmere goats (Erlangshan type), Bayes A and Bayes B achieved significantly higher genomic prediction accuracy than GBLUP and ssGBLUP for five economically important traits—fiber diameter, cashmere yield, cashmere thickness, cashmere length, and body weight—with bias estimates closer to 1 [12]. The core idea is to use high-density single nucleotide polymorphism (SNP) genotypes and phenotypic records to construct linear prediction models that increase genetic gain and selection efficiency [13,14,15].

In our previous study, we constructed a genomic reference panel for Alxa-type cashmere goats and evaluated the effects of genomic selection for cashmere fiber diameter and cashmere yield using ABLUP, GBLUP, and ssGBLUP in this population [16]. However, that study was limited to cashmere traits and did not compare the Bayesian series of models. Building on this foundation, the present study first imputed the genotype data of Alxa-type cashmere goats based on the previously constructed reference panel, and then extended the evaluation from cashmere fiber diameter and cashmere yield to body weight. Under a common cross-validation scheme, we systematically compared the estimation and prediction performance of Bayes A, Bayes B, and Bayes C in terms of both predictive ability and regression-based unbiasedness, and determined the non-genetic fixed effects to be included in the prediction model for each trait, with the aim of providing a scientific basis and technical support for precision breeding in this breed.

## 2. Materials and Methods

### 2.1. Animals and Sample Collection

Jugular venous blood was collected from 491 healthy Alxa-type cashmere goats (40 males and 451 females) at the Alxa Cashmere Goat Breeding Farm. Sequencing was performed at 1× depth, and the data were imputed to 10× using the previously constructed Alxa-type cashmere goat imputation reference panel (average imputation accuracy of 97.90%) for subsequent analyses.

### 2.2. Phenotypic Data

The traits investigated were cashmere yield, cashmere fiber diameter, and body weight. The phenotypic dataset comprised records from 491 individuals collected from 2021 to 2024, including 484 body weight records, 489 fiber diameter records, and 491 cashmere yield records measured at two years of age. Phenotypic records outside the mean ± 3 standard deviations were removed, and the quality-controlled data were used for subsequent analyses.

### 2.3. Genotypic Data

#### 2.3.1. Sequencing

Genomic DNA was extracted from the blood samples of Alxa-type cashmere goats. After quality control, the DNA was fragmented, end-repaired, and adenylated at the 3′ ends; DNBseq-specific adapters were ligated, and a DNA library was constructed by PCR enrichment. The double-stranded target-region library was then denatured, circularized, and digested to obtain single-stranded circular DNA, which was amplified by rolling-circle amplification to produce DNA nanoballs (DNBs) [17]. After library construction, quantification and quality control were performed with a Qubit instrument, and qualified libraries were sequenced.

The prepared DNBs were loaded onto a microarray chip and sequenced using combinatorial probe-anchor synthesis (cPAS). After polymerization of the sequencing primer anchor molecules and fluorescent probes on the DNBs, a high-resolution imaging system was used to capture, read, and recognize the optical signals, yielding single-base sequence information; repeated cycles generated the raw sequencing data.

#### 2.3.2. Quality Control of Sequencing Data

After sequencing, fastp was used to remove adapters and low-quality reads from the raw sequencing data, producing clean reads for subsequent analyses [18]. The filtering criteria were as follows: (1) reads containing adapter sequences were removed; (2) reads in which the N content exceeded 1% of the read length were removed; (3) reads in which low-quality bases (Q ≤ 5) accounted for more than 50% of the read length were removed; and (4) reads shorter than 100 bp were removed.

#### 2.3.3. Alignment to the Reference Genome

Clean reads were aligned to the reference genome (Goat.final.genome.fa) using BWA (version 0.7.17-r1188), and SAMtools (version 1.20) was used to calculate sequencing depth and genome coverage for each sample, in preparation for variant detection [19,20].

#### 2.3.4. SNP Detection and Annotation

Variant detection was performed on the processed files using the HaplotypeCaller module of GATK (version 4.18), and detected variants were filtered with VariantFiltration [21]. The filtering criteria included: (1) hard filtering based on quality scores (QD < 2.0; FS > 60.0; MQ < 40.0; SOR > 3.0; MQRankSum < −12.5; ReadPosRankSum < −8.0); (2) site-specific filtering (clusters of ≥2 SNPs within 5 bp; SNPs within 5 bp of an Indel); and (3) genotype quality filtering (sites with GQ < 20.0 were flagged as lowGQ) [22]. The final SNP set was functionally annotated using ANNOVAR (2016-02-01) to identify intronic, exonic, intergenic, 5′-UTR, 3′-UTR, splicing, upstream, and downstream variants [22].

#### 2.3.5. Genotype Imputation

Low-depth sequencing data were imputed using GLIMPSE2 based on the constructed reference panel, in four main steps:

(1) Chromosome chunks were defined: the chunk module of GLIMPSE2 was used to divide each chromosome into segments, with both the window size and the overlap buffer set to 0.1 cM.
(2) Reference haplotypes were built for each chunk: for each segment, the split-reference module of GLIMPSE2 was used to extract the corresponding reference haplotypes from the reference panel and convert them to BCF format.
(3) Low-depth data were imputed: the phase module of GLIMPSE2 was used to compare the segmented low-depth data with the reference haplotypes using a hidden Markov model, with five threads computing in parallel for haplotype inference and missing-genotype imputation; the final results were saved in BCF format.
(4) The imputed genotypes were converted to VCF or PLINK format: BCFtools was used to convert the BCF results to VCF format, which could then be further converted to PLINK format using PLINK.

After imputation, linkage disequilibrium pruning was performed using PLINK (v1.9) with the parameters—indep-pairwise 50 10 0.2 [23].

### 2.4. Genomic Selection

#### 2.4.1. Determination of Fixed Effects

The fixed effects considered in this study were sex (males and females; 2 levels), measurement year (2021–2024; 4 levels), maternal age (2–7 years; 6 levels), and group (groups 1–4 and the buck group; 5 levels). A general linear model linking phenotypes to the fixed effects was fitted, and the GLM procedure of SAS 9.2 software was used to determine the fixed effects affecting each trait, with the statistical significance of each fixed effect assessed by the F-test. The fixed effects influencing cashmere fiber diameter, cashmere yield, and body weight of Alxa-type cashmere goats were thus determined, and the significant effects were retained in the final model for analysis. The general linear model was

(1) y=μ+Si+Yj+Mk+Gm+en

where *y* is the phenotypic value of the trait; *μ* is the overall mean of the trait; *Si* is the effect of sex; *Yj* is the effect of measurement year; *Mk* is the effect of maternal age; *Gm* is the effect of group; and *en* is the random residual.

#### 2.4.2. Bayesian Models for Breeding Value Estimation

Based on pedigree, phenotypic, and genotypic data together with the fixed effects, a single-trait Bayesian linear model was established as follows:

(2) $$y=X\beta +\sum_{k=1}^{m}Z_{k}\mu_{k}+e$$

where *y* is the vector of corrected phenotypic values; *β* is the vector of fixed effects, including sex, measurement year, group, and maternal age, with design matrix *X*; $\mu_{k}$ is the random effect of the *k*-th SNP; $Z_{k}$ is the incidence matrix for the genotype coding (0/1/2) of the k-th SNP; and *e* is the vector of random residual effects.

Breeding values for cashmere fiber diameter, cashmere yield, and body weight were estimated using the Bayes A, Bayes B, and Bayes C methods. All Bayesian analyses were implemented using the BGLR package (version 1.1.0) in R (version 4.2.0). Parameters were estimated via Markov chain Monte Carlo (MCMC), with the number of iterations set to nIter = 12,000 and the burn-in period set to burnIn = 2000; all other parameters were left at the default settings of the BGLR package.

Bayes A assumes that each SNP has a distinct effect variance and that the effect variances follow an inverse chi-square distribution, where

(3) $$\mu_{k}|\sigma_{k}^{2}\sim N(0,\sigma_{k}^{2});\sigma_{k}^{2}\sim \chi^{2}(\nu ,S).$$

Bayes B assumes that only a proportion (1 − π) of SNPs have non-zero effects and that the non-zero effect variances follow an inverse chi-square distribution, where

(4) $$\mu_{k}|\sigma_{k}^{2},\pi \sim \left\{\begin{array}{cc} 0, & \mathrm{with}\;\mathrm{probability}\;\pi \\ N(0,\sigma_{k}^{2}), & \mathrm{with}\;\mathrm{probability}\;(1-\pi ) \end{array}\right.,\sigma_{k}^{2}\sim \chi^{-2}(\upsilon ,S).$$

Bayes C assumes that only a proportion (1 − π) of SNPs have non-zero effects and that all non-zero effects share a common variance, where

(5) $$\mu_{k}|\sigma^{2},\pi \sim \left\{\begin{array}{cc} 0, & \mathrm{with}\;\mathrm{probability}\;\pi \\ N(0,\sigma^{2}), & \mathrm{with}\;\mathrm{probability}\;(1-\pi ) \end{array}\right.,\sigma^{2}\sim \chi^{-2}(\upsilon ,S).$$

#### 2.4.3. Evaluation of Predictive Ability and Unbiasedness of Breeding Value Prediction

A five-fold cross-validation strategy was used to evaluate the genomic prediction performance of individual traits in Alxa cashmere goats, as follows: (1) the raw data were randomly divided into five subsets; (2) in each round, one subset was used as the validation set (with phenotypes removed), and the remaining subsets were used as the training set; (3) the training population was used to predict the breeding values (GEBVs) of the test population; (4) the correlation and regression coefficients between the GEBVs and the corrected phenotypes were calculated; and (5) the procedure was repeated five times.

Predictive ability: the Pearson correlation coefficient between the predicted breeding values (${GEBV}_{test}$) and the corrected phenotypic values ($y_{test}^{*}$) of the validation population, representing the correlation between two continuous variables [24]. The formula was

(6) $$r(y_{test}^{*},{GEBV}_{test})=\frac{cov(y_{test}^{*},{GEBV}_{test})}{var(y_{test}^{*})var({GEBV}_{test})}$$

where $r(y_{test}^{*},{GEBV}_{test})$ is the covariance between ${GEBV}_{test}$ and $y_{test}^{*}$, $var(y_{test}^{*})$ is the variance of $y_{test}^{*}$, and $var({GEBV}_{test})$ is the variance of ${GEBV}_{test}$.

Unbiasedness: the regression coefficient of the corrected phenotypic values on the predicted breeding values of the validation population; values closer to 1 indicate that the predicted breeding values are unbiased predictions of the corrected phenotypes [24].

## 3. Results

### 3.1. Phenotypic Data

Phenotypic records of body weight, cashmere fiber diameter, and cashmere yield were collected from 491 Alxa-type cashmere goats and subjected to descriptive statistics; all three traits followed a normal distribution (Figure 1). The mean body weight was 32.29 ± 5.38 kg with a coefficient of variation (CV) of 16.67%; the mean cashmere fiber diameter was 14.34 ± 0.81 μm with a CV of 5.64%; and the mean cashmere yield was 771.09 ± 144.08 g with a CV of 18.69%. These results indicate that the fiber fineness of the population was relatively uniform with good quality stability, whereas body weight and cashmere yield showed relatively large between-individual variation (Table 1).

### 3.2. Genotypic Data

The average number of raw reads was 21.56 M, and the average number of clean reads after quality control was 21.23 M. The reference panel achieved an average imputation accuracy of 0.979. Detailed information on the imputed genotype data and the reference panel is available in our previous study [16]. The average alignment rate of the sequencing data was 99.74%, indicating normal alignment results that satisfied the requirements for subsequent variant detection and related analyses. The reference genome used was the Inner Mongolia cashmere goat reference genome constructed previously by our group (Goat.final.genome.fa). After imputation and removal of linked loci, 1,132,679 loci remained for subsequent analysis.

### 3.3. Genomic Selection

#### 3.3.1. Fixed Effects

As shown in Table 2, measurement year had a highly significant effect (*p* < 0.01) on body weight, cashmere fiber diameter, and cashmere yield; sex had a highly significant effect (*p* < 0.01) on body weight and cashmere yield but no significant effect on cashmere fiber diameter (*p* > 0.05); maternal age significantly affected body weight (*p* < 0.05) but had no significant effect on fiber diameter or cashmere yield (*p* > 0.05); and group had a highly significant effect on all three traits (*p* < 0.01). These factors were therefore included as fixed effects in the genetic model to reduce systematic errors caused by environmental factors and sex differences, thereby improving the accuracy of the genetic model.

#### 3.3.2. Evaluation of Predictive Ability and Unbiasedness of Breeding Value Prediction

The predictive ability of estimated breeding values for body weight, cashmere fiber diameter, and cashmere yield in Alxa-type cashmere goats was evaluated using the five-fold cross-validation strategy; the results are shown in Table 3 and Table 4. For body weight and cashmere fiber diameter, Bayes B achieved the highest predictive ability whereas for cashmere yield, Bayes A achieved the highest predictive ability, although the difference between Bayes A and Bayes B was small. For unbiasedness, Bayes A and Bayes B gave the same value for body weight, and Bayes B was closest to 1 for cashmere fiber diameter and cashmere yield.

## 4. Discussion

### 4.1. Effects of Non-Genetic Factors on Economic Traits

The body weight, cashmere fiber diameter, and cashmere yield of cashmere goats are influenced by many factors, such as individual age, maternal age, measurement year, group/farm, and sex [25,26,27,28]. Sex had a highly significant effect on body weight and cashmere yield but no significant effect on cashmere fiber diameter, which may be related to the physiological development stage of two-year-old animals: (1) testosterone secreted by the testes of bucks is one of the main growth-promoting hormones and can significantly promote bone growth, muscle protein synthesis, and red blood cell production, resulting in significantly higher growth rates and adult body weights in bucks than in does, which in turn drives higher cashmere yield [29]; (2) cashmere fiber diameter is primarily governed by follicle type and genetic factors, is markedly modulated by non-genetic factors such as age, but is relatively insensitive to sex-related factors, and therefore exhibits no significant sex difference; and (3) the large difference in sample size between bucks and does may have influenced the statistical results.

Measurement year had a highly significant effect on all three traits, indicating that differences in environmental factors such as climatic conditions, feeding management, and pasture nutrition among years significantly affect the main economic traits of cashmere goats. This result is consistent with previous studies and reflects the combined effects of annual variation in precipitation, temperature, pasture resources, and feed supply on animal growth, development, and cashmere production [30].

Group also had a highly significant effect on all three traits, consistent with previous findings, reflecting differences among flocks in pasture utilization (actual grazing areas differed among groups, with uneven natural pasture yield and forage nutrition), population structure (age, sex, and strong/weak composition), and management practices [26,31]. These results indicate that, in practical breeding work, independent flock records must be established, and group effects must be corrected when performing joint genetic evaluation or comparing phenotypic levels across groups, to avoid biases caused by management differences. The highly significant group effect also suggests that optimizing feeding management and standardizing technical protocols are important for fully exploiting the genetic potential of the population.

Maternal age significantly affected body weight but not cashmere fiber diameter or cashmere yield. The main possible reasons are: (1) maternal age regulates the nutritional intake and growth of offspring by influencing lactation capacity, maternal behavior, colostrum quality, and the ability to allocate nutrients appropriately to the fetus, resulting in significant differences in two-year-old body weight among offspring of does of different ages [32]; and (2) cashmere fiber diameter and cashmere yield are mainly regulated by genetic factors and photoperiod, and the density and structure of secondary hair follicles are largely fixed during the embryonic stage, making them insensitive to maternal age [33]. The potential influence of maternal age should continue to be considered in future breeding evaluations.

Based on the above analyses, the three major economic traits in this study—body weight, cashmere fiber diameter, and cashmere yield—differed markedly in their influencing factors and interrelationships. In terms of factor effects, body weight was significantly affected by sex, measurement year, group, and maternal age; cashmere yield was mainly affected by sex, measurement year, and group; and fiber diameter, as a trait reflecting cashmere quality, was mainly regulated by group and measurement year, showing relatively low sensitivity to sex and maternal age. Sex mainly promoted body weight gain in rams, which in turn drove an increase in cashmere yield, while its effect on fiber diameter was limited. The common significant effects of measurement year and group on all three traits mainly reflected the combined changes in interannual climatic conditions, natural pasture yield and nutrition, and feeding management levels, which directly or indirectly affected the growth and development of goats and cashmere production; these effects may also have indirectly influenced cashmere yield and fiber growth through mediators such as body weight and nutritional status. Accordingly, in practical breeding, body weight can be used as an auxiliary selection indicator to indirectly improve cashmere yield, while fiber fineness should be controlled separately as an independent quality trait; moreover, when establishing a genetic evaluation model, the corresponding fixed effects should be corrected separately according to the characteristics of each trait.

### 4.2. Predictive Ability and Unbiasedness of Genomic Breeding Value Estimation

This study compared the genomic prediction performance of Bayes A, Bayes B, and Bayes C for three important economic traits in cashmere goats—body weight, cashmere fiber diameter, and cashmere yield—and evaluated the effects of different prior assumptions on the predictive ability and unbiasedness of genomic selection.

Predictive ability is jointly constrained by the heritability (h^2^) of the trait, the degree of linkage disequilibrium (LD) between markers and traits, and the genetic architecture [34]. From the predictive ability results, the accuracy estimates of the three methods across the three traits fluctuated within a narrow range, and no single method showed an absolute advantage. The predictive ability for body weight was slightly lower than that for cashmere fiber diameter and cashmere yield. This difference may originate from the genetic characteristics of the traits themselves: body weight is a typical low-heritability trait (strongly affected by environmental disturbances such as feeding management, season, and maternal effects), whereas cashmere fiber diameter and cashmere yield have moderate to high heritability and are more readily captured by molecular markers [35]. It should be noted, however, that heritability is population-specific. The present study did not directly estimate the heritability of the corresponding traits in this cashmere goat population, and the above explanation is based mainly on general expectations from the existing literature. Therefore, the differences in predictive ability cannot be directly attributed to heritability on this basis. The three Bayesian methods showed no marked differences in predictive ability, indicating that, at the marker density, sample size, and genetic structure of this cashmere goat population, the choice of prior distribution has a limited effect on improving the accuracy of genomic breeding value estimation [8].

The unbiasedness estimates of the three methods were also relatively close, ranging from 0.609 to 0.816. For body weight, the unbiasedness values of Bayes A and Bayes B were higher than that of Bayes C, indicating that Bayes A or Bayes B can effectively reduce prediction bias when studying traits strongly influenced by environmental and maternal effects. For cashmere fiber diameter and cashmere yield, Bayes B showed the highest unbiasedness, whereas Bayes C was again the lowest. Bayes C showed lower unbiasedness than the other two methods for all traits, suggesting a more pronounced tendency of its predictions to shrink toward the mean—that is, the variability of the predicted breeding values was relatively compressed. This is better described as a shrinkage phenomenon rather than simple overall underestimation. Meuwissen et al. showed that BLUP assuming equal variance for each 1 cM segment achieved an accuracy of 0.73, whereas Bayesian methods assigning variance priors to each segment increased the accuracy to 0.85 [4]. Notably, Bayes C adopts exactly this equal-variance approach. However, the present study does not directly demonstrate that the prior variance structure is the direct cause of the above differences; accordingly, we present the difference in prior specification of Bayes C only as one possible explanation: unlike Bayes A and Bayes B, which assign a specific effect variance to each locus, Bayes C assumes that all loci included in the model share one common variance and therefore cannot achieve the locus-specific shrinkage of effects of different sizes that Bayes A and Bayes B provide. This may partly explain its poorer unbiasedness. At the same time, these differences may also be related to factors such as training population structure, marker density, and trait genetic architecture, and the present study cannot discriminate among these factors.

Body weight was the trait with the largest number of significant non-genetic effects in the fixed-effect analysis (measurement year, sex, maternal age, and group; Table 2), while also showing the relatively lowest predictive ability (Table 3). This is consistent with the expectation that a larger non-genetic component leaves a smaller proportion of phenotypic variation to be captured by markers. In contrast, only measurement year and group were significant in the fixed-effect structure of fiber diameter, which also showed the relatively highest predictive ability. The regression coefficients showed the same ranking, with the largest differences among the three methods observed for body weight (0.641–0.800; Table 4) and the smallest for fiber diameter (0.609–0.713). Taken together, the three traits exhibited a consistent pattern: traits with a larger non-genetic contribution had lower predictive ability and greater differences among methods. We present this as an observation from the data that merits testing in larger populations and different genetic backgrounds, rather than as an established relationship, since the three traits also differ in other respects, including their genetic architecture and the accuracy of phenotypic records.

In this dataset, Bayes B showed a slightly more favorable overall balance between predictive ability and regression-based unbiasedness.

### 4.3. Limitations

This study has several limitations: (1) The population size for genomic prediction was relatively small (491 animals). Although it was sufficient to compare the predictive ability of different prior specifications at the population level, it limited the precision of the estimates and their statistical separation. (2) The sex ratio in the population was imbalanced (40 males and 451 females). Although sex was included in the model as a fixed effect, this imbalance weakened the evaluability of sex-specific prediction and may have reduced the contribution of male individuals to the estimation of marker effects. (3) All individuals came from a single breeding farm, and the validation set was partitioned based on the whole population; therefore, the reported predictive ability may be overestimated when applied across environments or genetic backgrounds.

## 5. Conclusions

In summary, non-genetic factors significantly affect the main production traits of cashmere goats. When constructing genomic selection genetic evaluation models for cashmere goats, fixed effects should be set appropriately according to the non-genetic effect structure of each trait to improve the reliability of breeding value estimation. Breeding values for body weight, cashmere fiber diameter, and cashmere yield were estimated using Bayes A, Bayes B, and Bayes C, and the three methods were generally comparable in this population.

## Figures and Tables

**Figure 1:**
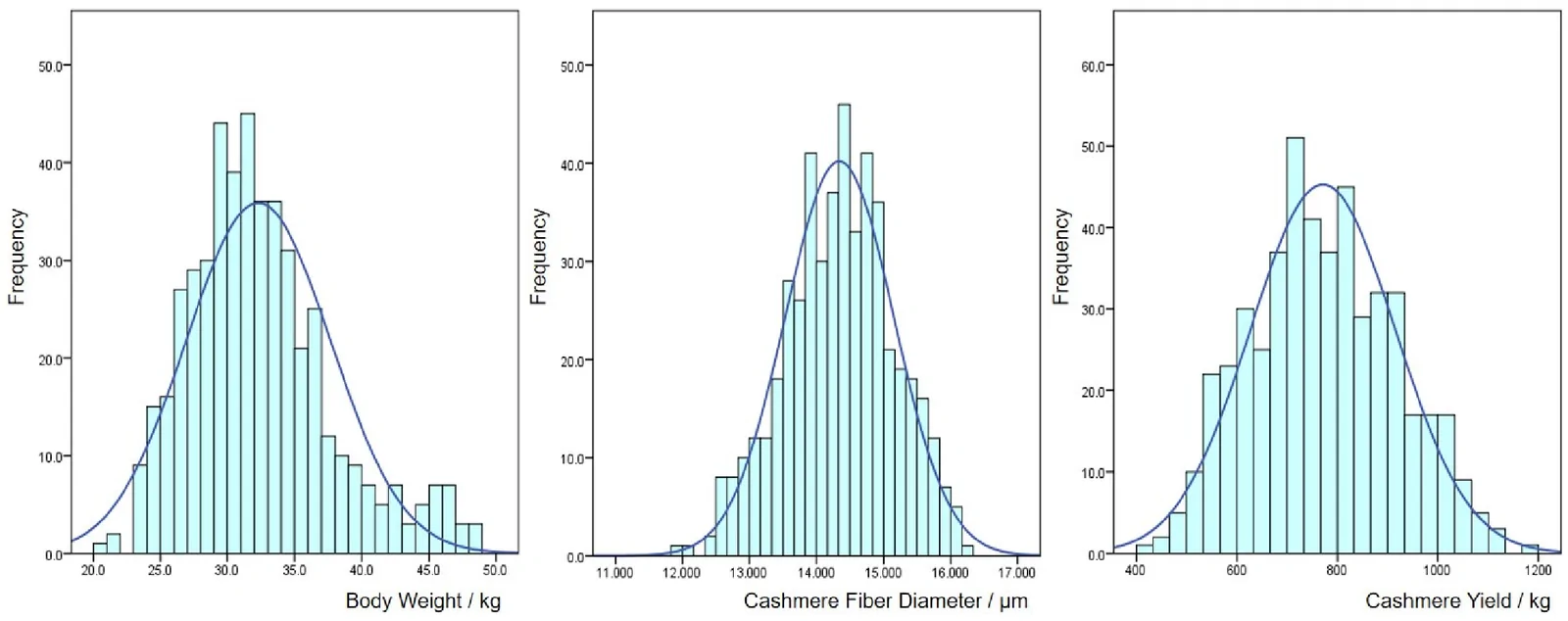
Normal test results.

**Table 1:** Basic statistics of phenotypic records for Alxa-type cashmere goats.

Trait	Sample Size	Mean	Min	Max	SD	CV
Body weight (kg)	484	32.29	20.80	48.00	5.38	16.67%
Cashmere fiber diameter (μm)	489	14.34	11.97	16.27	0.81	5.64%
Cashmere yield (g)	491	771.09	425.00	1180.00	144.08	18.69%

**Table 2:** Tests of fixed effects.

Trait	Fixed Effect	DF	Sum Sq	Mean Sq	F-Value	*p*-Value
Body weight (kg)	Measurement year	4	4897	1224.1	60.97	<0.01
	Sex	1	5956	5956	335.2	<0.01
	Maternal age	5	434	86.77	2.952	<0.05
	Group	4	6096	1524	86.64	<0.01
Cashmere fiber diameter (μm)	Measurement year	4	83.83	20.956	42.03	<0.01
	Sex	1	1.6	1.5898	2.395	>0.05
	Maternal age	5	3.8	0.7559	1.137	>0.05
	Group	4	29.31	7.327	12	<0.01
Cashmere yield (g)	Measurement year	4	965,210	241,303	12.76	<0.01
	Sex	1	144,963	144,963	7.082	<0.01
	Maternal age	5	49,704	9941	0.477	>0.05
	Group	4	359,845	89,961	4.464	<0.01

Note: *p* < 0.01, highly significant; *p* < 0.05, significant; *p* > 0.05, not significant.

**Table 3:** Predictive ability evaluation results.

Trait	Bayes A	Bayes B	Bayes C
Body weight (kg)	0.185 ± 0.104	0.187 ± 0.101	0.185 ± 0.103
Cashmere fiber diameter (μm)	0.298 ± 0.077	0.299 ± 0.078	0.291 ± 0.078
Cashmere yield (g)	0.241 ± 0.082	0.240 ± 0.081	0.237 ± 0.085

**Table 4:** Evaluation results of unbiasedness.

Trait	Bayes A	Bayes B	Bayes C
Body weight (kg)	0.800 ± 0.306	0.800 ± 0.310	0.641 ± 0.256
Cashmere fiber diameter (μm)	0.681 ± 0.332	0.713 ± 0.369	0.609 ± 0.291
Cashmere yield (g)	0.788 ± 0.228	0.816 ± 0.241	0.722 ± 0.220

## Data Availability

The data that support the findings of this study are available on request from the corresponding author. The data are not publicly available due to privacy concerns and to protect the confidentiality of participants.
